# Subarachnomegaly—venous congestion of infancy

**DOI:** 10.1007/s00381-021-05328-z

**Published:** 2021-10-23

**Authors:** Laura V. Sainz, Martin U. Schuhmann

**Affiliations:** 1 Institute of Neuroinformatics, ETH, Zürich, Switzerland; 2 Division of Pediatric Neurosurgery, Department of Neurosurgery, University Hospital of Tuebingen, Tübingen, Germany

**Keywords:** Subarachnomegaly, Macrocephaly, Cortical subarachnoid space, Venous sinuses, MR phlebography

## Abstract

**Purpose:**

Multiple names within the literature refer to a clinical picture affecting infants and consisting of a large or fast growing head circumference with enlarged cortical subarachnoid spaces (CSAS) while cranial sutures are open. This myriad of terms demonstrates the confusion about the entity, that may even group together different etiological processes. In this review, we aim to shed light on this matter in an effort to restate the defining features of the clinical picture and sum the evidence and current understanding of its pathophysiology and related imaging findings.

**Methods:**

Extensive and updated review of the literature with special focus on defining features, clinical history with long term evaluation and pathophysiological process.

**Results:**

Functional and molecular CSF studies as well as clinical evidence challenges the common pathophysiological theory based on non-functional arachnoid villi. Conversely, there is increasing evidence supporting cerebro-venous system abnormalities as the main pathophysiological factor. Additionally, long term cohorts studies show that it may have subtle but irreversible neurodevelopmental consequences.

**Conclusion:**

Subarachnomegaly is an age-related condition of the infancy with radiological enlargement of CSAS and often self limiting course. However, considering the evidence on pathophysiology as outlined herein and long term outcome reports, further research effort is needed to assess the consequences of venous outflow impairment and enlarged CSAS and how this relates to imaging findings and neurodevelopment test results later in life

## Introduction and terminology

Many different terms used in literature refer to this entity include benign external hydrocephalus, benign enlargement of subarachnoid space, extra-ventricular hydrocephalus, subdural effusions of infancy, pseudo-hydrocephalus, benign extra-axial collection of infancy, benign subdural effusions in infants, subdural hygroma, pseudohydrocephalus, benign communicating hydrocephalus, and extra-ventricular obstructive hydrocephalus [1–3].

The main clinical feature of the entity is the development of macrocephaly presenting with a mean age of 6–7 months at onset with radiological enlargement of the cortical subarachnoid spaces, termed subarachnomegaly [4]. Previous to the advent of MRI, the subarachnoid spaces were difficult to distinguish from the subdural space; therefore, subdural descriptive terms have also been used to refer to this entity. However, nowadays, diagnosis is based purely on cortical subarachnoid space enlargement, and subdural collections without subarachnomegaly exclude the diagnosis [5, 6]

Another important hallmark is that there is typically mild or no ventriculomegaly, and signs of classic obstructive hydrocephalus are absent [2]. In this regard, the terms external or extra-ventricular hydrocephalus may be misleading, since the term hydrocephalus is linked to ventriculomegaly by many. Moreover, increasing evidence challenges the traditional hypothesis of impaired CSF absorption by immature non-functional arachnoid villi as the main underlying cause.

Many papers refer to subarachnomegaly as a self-limiting and benign entity [7, 8] since in the majority of cases, the enlargement of the subarachnoidal spaces subsides without apparent developmental or neurological consequences. However, only very few studies have been assessing the long-term developmental and neurological status of these patients. Hence, this apparently benign course may just be because long-term outcome has not been evaluated thoroughly with developmental tests [9]. A terminology including “benign” should be avoided since it assumes an unproblematic outcome, a fact that is still under research. Indeed, in some studies, a non-negligible proportion of patients presented subtle neurocognitive difficulties and delayed motor skills [1, 10].

In summary, subarachnomegaly comprises macrocephaly as the main clinical finding and enlargement of subarachnoidal spaces as the radiologically defining feature. In this work, we review radiological findings, clinical course, and recent evidence regarding etiology that supports the hypothesis of venous congestion as the principal pathophysiological factor in developing subarachnomegaly.

## Diagnostic criteria

- Macrocephaly > 95th percentile, at the expense of enlarged cortical subarachnoid space [1].
- Predominantly frontal subarachnomegaly with mild or no ventriculomegaly [2],
- Manifestation in infants 6–18 months of age, predominantly when sutures are open [11, 12]
- Exclusion of classic hydrocephalus and related pathologies e.g. tethered cord [13]
- Exclusion of hypomagnesemia, mucopolysaccharidosis, achondroplasia, agenesis of corpus callosum, Sotos syndrome and hyperglutaremia [1]

## Clinical course

The typical clinical presentation begins with the referral of an otherwise normal infant because of a fast-growing head with crossing percentiles. To consider macrocephaly, head circumference must be two standard deviations above the mean for age and sex, as described in a standard growth chart. Typically, head circumference at birth is normal [11, 14] or slightly higher than normal [15, 16].

The initial radiological assessment reveals enlarged subarachnoid space and no or mild ventriculomegaly. Subarachnomegaly is the most common cause of macrocephaly in infancy [9, 17, 18]. In some studies, family history of macrocephaly has been reported [17, 19]. However, it is still unclear if there is a truly familial component. Major neurological deficits are usually not present, but in some cases, mild motor and language delay have been noted, although final developmental status is often reported as normal [1].

**Fig. 1:**
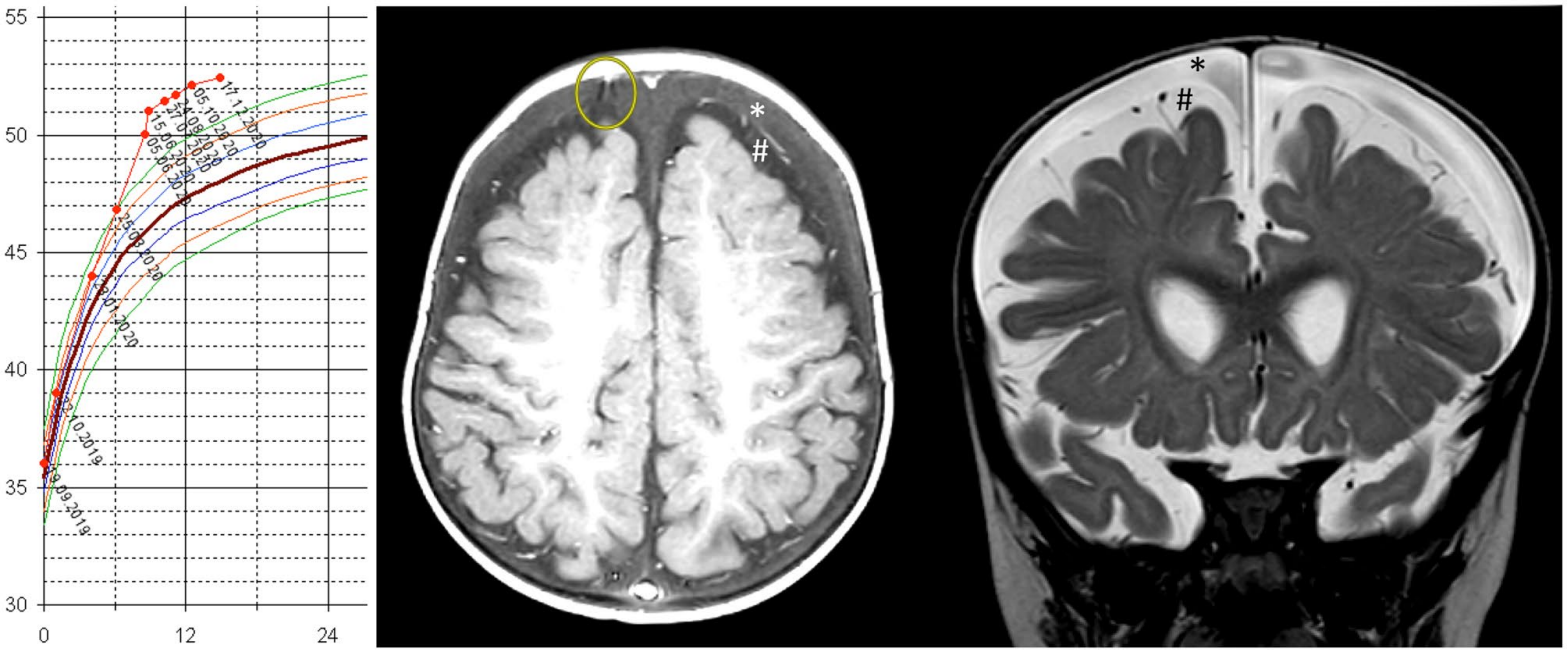
10-month-old boy presenting with a slowly rising head circumference visible at 6 months of age when reaching the 97th percentile (P97). At 8 months of age, his head was 2 cm above P97; the child was without symptoms. Within 10 days, his head circumference rose by 1 cm, and he showed signs and symptoms of raised intracranial pressure in with increasing severity (left picture). The MRIs (middle: axial T1 plus contrast and right: coronal T2) show an expanded subdural fluid space (*) above the expanded plus enlarged subarachnoid spaces (#) containing vessels and arachnoid membranes. The fact that the subarachnoid space is not compressed by the subdural fluid on top indicates that there is pressure equilibrium between both compartments. The yellow circle in the T1-weighted axial MRI depicts a bridging veins stretched by the expansion of the subdural space

A wide corpus of papers relates subarachnomegaly with an increased risk of developing subdural hematoma. (Fig. 1) It is considered to be the most common complication in these infants after minimal or even without head trauma [1, 11]. However, non-accidental trauma should also be excluded in these patients. Simulations predict this increased risk by modeling the stretch to which the cortical bridging veins are subjected to by the enlargement of the cortical subarachnoid space [20].

**Fig. 2:**
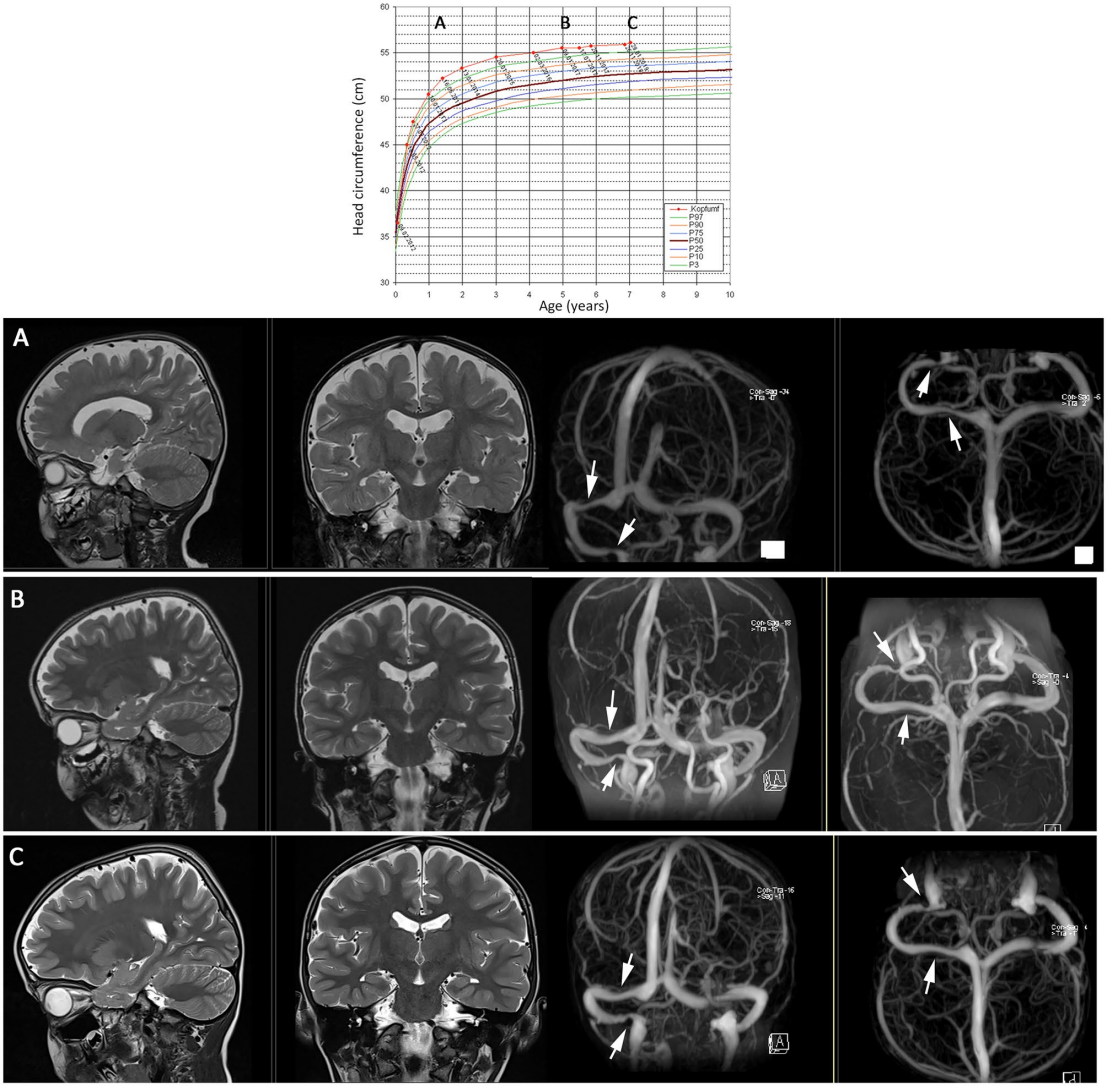
Typical course of head circumference (HC) and corresponding MRI controls during follow-up showing a slowly regressing CSAS in a persisting macrocephalic child with subarachnomegaly and venous sinus pathology. The top shows the head circumference curve over 7 years. The boy started with a HC at P50 at birth and developed a HC at P97 at 6 months of age. **A** At 1 y and 5 months, his HC was ~ 1 cm above P97 and the first MRI was performed. The ventricles are slightly larger without fulfilling the criteria for hydrocephalus. The frontal and parietal CSAS are enlarged, and the contrast-enhanced 3D venography shows a > 50% narrowing of the right transverse sinus, which was the direct drainage continuation of the sagittal sinus and a > 75% stenosis of the right sigmoid sinus immediately before the jugular foramen (white arrows). He maintained from that time point on his HC curve position about 1 cm above P97. **B** At age of 5 years, his CSAS have become smaller but are still wider than normal. The right transverse sinus stenosis has disappeared, and the pre-jugular foramen stenosis has remained unchanged. **C** The sinus situation is unchanged at age of 7 years; however, the l CSAS have decreases a little bit further

The majority of cases with subarachnomegaly are followed and observed without intervention as the radiological widening of the subarachnoid space progressively subsides spontaneously within 1–2 years [13]. Head circumference normally stabilizes on a course parallel but above P97 before 18 months of age. In some cohorts, more than half of infants remain macrocephalic throughout the follow-up period [12, 19] (Fig. 2).

## Imaging findings

**Fig. 3:**
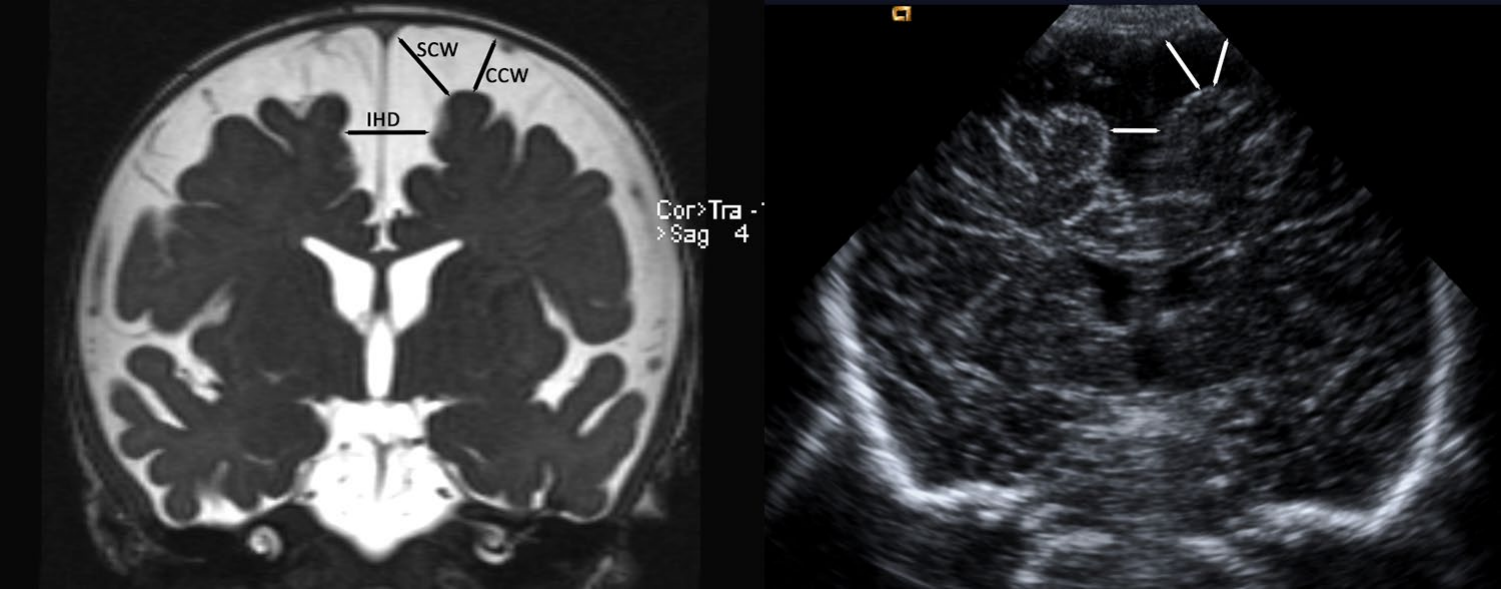
Radiological parameters to assess CSAS width on MRI and US scans from the same boy at an age of 5.5 months, when he had developed macrocephaly 2 cm > P97 and very pronounced subarachnomegaly. The underlying cause was a high-grade bilateral transverse sinus stenosis. A coronal T2-weighted MRI scan (left) and a coronal transfontanellar ultrasound scan (right) are shown. The craniocortical width (CCW), the sinocortical width (SCW), and the interhemispheric distance (IHD) are shown correspondingly in MRI and ultrasound

The study of a macrocephalic infant usually begins with an ultrasound through the anterior fontanel. An increased cortical subarachnoid space with normal or mildly enlarged ventricles is used as a diagnostic criterion. Subarachnomegaly is predominantly frontal and is assessed via three main measurements: sinocortical width (SCW), craniocortical width (CCW), and interhemispheric distance (IHD) (Fig. 3). Normal ranges for CCW, SCW, and IHD are from 4 to 10 mm, from 2 to 10 mm, and from 6 to 8.5 mm, respectively [21, 22]. Typical findings for active, obstructive hydrocephalus as ventricular enlargement and periventricular lucency are absent.

Ultrasound may not be sufficient to exclude other pathologies or distinguish subdural collections from widened CSAS or cerebral atrophy. If possible, a CT scan should be avoided in this early phase of life. Therefore, MRI is used to complete the assessment or to address complications of a known case in the follow-up. Particularly, MRI has allowed distinguishing between subdural effusions or hematoma and enlargement of the cortical subarachnoid space, helping to define subarachnomegaly as a distinct entity (see Fig. 1). Subdural collections appear more hyperintense in T1, proton-density, or T2-weighted sequences and lack the flow void sign (corresponding to a vessel) that appears in subarachnoidal spaces [5]. Moreover, MRI allows thorough evaluation of anatomical structures excluding other pathologies as arachnoid cysts, obstructive hydrocephalus, or cerebral atrophy. Cerebral atrophy is distinguished from subarachnomegaly because the latter shows bifrontal and not diffusely prominent sulci [2, 4]. Available high-resolution T2-weighted MRI sequences allow for quantification of the cortical subarachnoidal space volume. In our earlier work, mean CSAS volume above the intercommisural plane in a cohort of 17 infants with subarachnomegaly was found to be 194.48 ml at diagnosis, predominantly in the frontal region, corresponding to above-normal measurements for CCW, SCW, and IHD [23]. Moreover, Cinalli et al. described in a retrospective series similar findings of a statistically significant increased CSAS volume in these patients as compared to controls [24].

Diffusion tensor imaging (DTI) provides quantitative information about the diffusion properties within white matter. Fractional anisotropy (FA) is a DTI parameter that measures the spatial differential restriction to diffusion. In classical hydrocephalic patients, FA is typically low. This reduction is thought to reflect microstructural changes such as demyelination or loss of axonal integrity as damage after sustained increase of intracranial pressure (ICP). Increased FA values, on the contrary, indicate a high restriction to diffusion. In a retrospective analysis of a cohort of 15 subarachnomegaly infants, FA values were increased with respect to healthy controls in the genu and splenium of the corpus callosum (gCC, sCC) normalizing during a mean follow-up of 14 months [25]. This fact is thought to be related to mechanical pressure on the white matter. However, it is still unclear how changes in these DTI parameters correlate with values of ICP and neurodevelopment statutes and outcomes.

MR-venography has also become very relevant in understanding subarachnomegalic infants. In a significant proportion of these patients, the cerebrovenous system is compromised. In a cohort of 17 patients, 15 (88%) presented with abnormal phlebographies of their main venous sinuses, ranging from hypoplastic to severe stenosis. Moreover, the number and degree of affected venous segments showed a significant positive correlation with the CSAS volume with a Pearson coefficient of 0.57 (*p* = 0.028) [23]. In their analysis of 97 patients, Cinalli et al. [24] confirmed venous abnormalities in 84.53% of patients in MR phlebography compared to 25.33% of prevalence in the control group (*p* < 0.001). Despite using a different scoring system to describe the severity of venous outflow impairment as compared to Sainz et al. [23], these authors also confirmed the positive correlation between severity of venous abnormalities and CSAS volume (*p* = 0.01) [24]

Reversible collapse of the sinuses has been encountered [23, 26], pointing toward the existence of not only fixed sinus wall abnormalities but also abnormal dynamics, similar to that observed in IIH adult patients [27].

## Pathophysiology

Many theories and different etiological factors have been described. The common theory for subarachnomegaly in infants suggests that it is produced by impairment of CSF absorption by immature arachnoid villi [11, 28]. Along similar lines, it has been hypothesized that the enlargement of the subarachnoid space may be caused by communicating hydrocephalus due to a distal (high convexity or parasagittal) block [29]. Others, consider subarachnomegaly as a variation in brain development with a transient accumulation of CSF due to a fast increasing head size, for instance in patients born prematurely with fast catch-up growth [30]. Other authors hypothesized that the underlying cause is a cephalocranial disproportion [31]. Lastly, subarachnomegaly has also been contemplated to be caused by a developmental disturbance of inadequate skull growth in relation to brain growth [32]. The most commonly cited immature arachnoid villi theory hypothesizes that a failure in CSF absorption would lead to dilation of cortical subarachnoid spaces because of a reabsorption deficit from the subarachnoid spaces. Congenital absence of these structures was postulated as an explanation [2, 33].

The widening of the CSAS at the expense of a growing skull with open fontanelles and sutures would prevent an increase of intracranial pressure. However, studies of arachnoid granulations reveal that villi immaturation or vili absence is a usual finding among all infants regardless of their CSF condition [34]. A primary CSF absorption problem, in the classical sense of communicating hydrocephalus, would lead predominantly to ventriculomegaly and not to an expansion of the CSAS.

Furthermore, the main CSF absorption pathway appears not to be through the arachnoid villi neither in adults [13] nor in infants [35]. Quantitative studies analyzing CSF flow conclude that the capillary bed of the deep white matter (capillary bed) must play an active role in CSF dynamics, especially relevant in infants, where the net aqueduct CSF flow is into the ventricles and not outward. Ventricular reflux is considered a normal feature in children less than 2 years old [27]. These facts challenge the traditional CSF bulk flow concept with a net outflow from the ventricles that would support the hypothesis of an impaired CSF absorption at the arachnoid villi as the main underlying cause for subarachnomegaly [27, 36]. Additionally, molecular and cellular studies have revealed physiological complex CSF circulation dynamics. At the core of the new concept is the Virchow Robin space, the anatomical unit where an active and regulated exchange of substances takes place in what has been called the third circulation acting as a clearance pathway for the interstitial fluid of the capillary bed within the tissue [37]. This regulated exchange depends, among other factors, on the gradient of hydrostatic pressure between blood stream (perforating vessels), CSF space, and neural tissue compartments. Taking into account the proportion of perforating vessels within CSF compartments, the CSAS arises as a functionally independent compartment from the ventricular space.

The CSAS becomes particularly relevant in regard to CSF dynamics in those clinical settings where ventricular size remains unchanged or only mildly increased as in infants with subarachnomegaly. In the cohort of Sainz et al. [23], 5/17 children (29%) had ventricles above normal (FOHR > 0.37) with a cohort mean of 0.36. Cinalli et al. confirmed this finding of a certain degree of ventricular enlargement [24].

Any form of cranial venous outflow congestion, as those shown in the MR-venography of a majority of subarachnomegalic patients, may result in an increased sinus venous pressure (venous hypertension) that will lead to a post-capillary rise of pressure. This, in turn, will increase the pressure within the capillary bed impairing also CSF reabsorption from the interstitial space. The subsequent increase in ICP (increased venous and increased CSF pressure) will promote an accelerated skull growth, eased by open sutures and fontanelles, creating a disproportion between the skull volume and the normal brain volume. This additional space is of course taken by the expanding CSAS. Added to the above-cited studies in subarachnomegalic children, there is mounting evidence of a relation between the venous system and CSF dynamics. Hydrocephalic infants exhibit high-pressure measurements in the venous system [38]. Achondroplasy, with known fixed venous sinus stenosis at the skull base, is related to the development of internal hydrocephalus and subarachnomegaly. Compromising venous outflow, even in acute scenarios, i.e., superior vena cava obstruction, may result in both subarachnomegaly and internal hydrocephalus [39, 40].

Moreover, direct changes in the jugular outflow, and hence, the pressure in the cerebral venous system, have been related to changes in the cortical subarachnoid space volume [33, 41]. In this regard, adult patients after a decompressive craniectomy, with equivalent less rigid skull analog to subarachnomegaly infants, frequently develop subarachnoid CSF collections. The volume of these cortical subarachnoid collections correlates positively with the extent of midline removal of bone over the sinuses and therefore proneness of the sagittal sinus to collapse under atmospheric pressure, an event that increases venous outflow resistance [42].

As described above, MR venogram abnormalities have been observed in subarachnomegalic patient cohorts or in case reports as an apparent common finding [23, 24, 27]. The prevalence of these MR venogram abnormalities in healthy infants appears to be less common and never severe [24], and it is most likely that the venous anomaly is related to the pathophysiology of CSAS enlargement. The correlation between the extent of venous sinus alterations and the CSAS volume was independently found in two studies with differences in the scoring system and was comparably significant and positive [23, 24]. These results support the hypothesis that the impairment of venous outflow (and thus an increased intracranial pressure) is indeed the relevant and driving factor in subarachnomegaly and first and foremost that it is related with its severity. On the other hand, dynamic and reversible venous stenosis due to collapsible sinuses has been described and reported to improve after CSF pressure reduction through shunt insertion in cases of CSF infection and subarachnomegalic patients, revealing that at least certain children present with a collapsible form of sinus stenosis [23, 26].

**Fig. 4:**
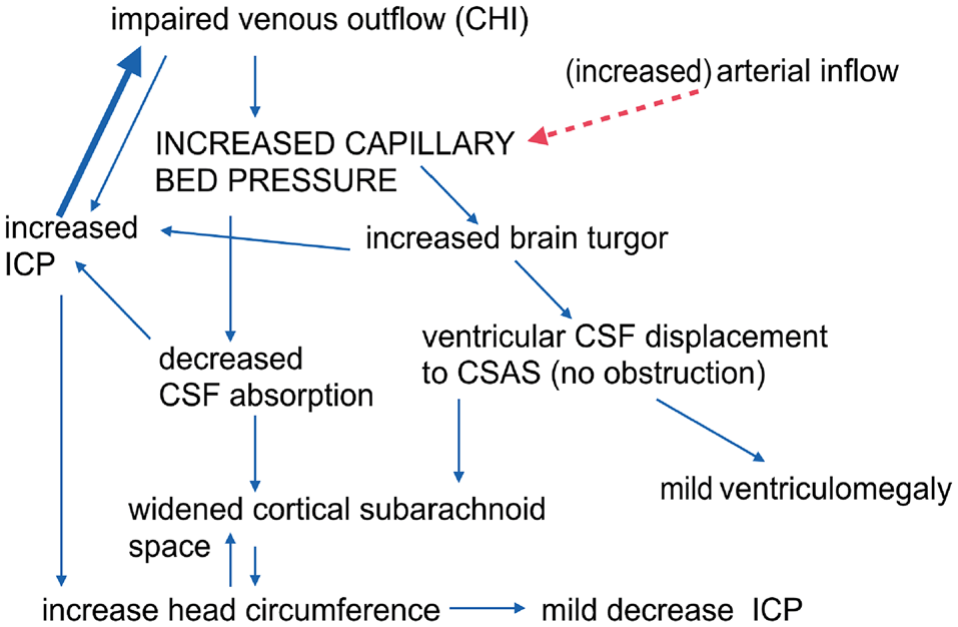
Pathophysiological cycle. An increased venous pressure with impaired venous outflow increases capillary bed pressure, which in turn decreases CSF absorption and increases ICP. The latter then aggravates venous stenosis (thick blue arrow). The pathological mechanism for other forms of subarachnomegaly with no MR phlebography anomalies remains elusive. They may correspond to cases with increased arterial inflow resembling the hyperemic form of pseudotumor cerebri in adults (red dashed arrow). (Adapted from [23])

Some subarachnomegalic infants, between 12 and 21% in the abovementioned phlebography studies, however, present with no MR-venography abnormalities. In these cases, a pre-capillary hypertension of the capillary bed is hypothesized corresponding to a hyperemic form of subarachnomegaly. An increased arterial inflow, instead of a venous stenosis, would end up raising capillary bed pressure producing consequently the same clinical picture [27]. There is some evidence supporting this mechanism in adults producing the less common form of hyperemic pseudotumor cerebri (as opposed to stenotic pseudo-tumor cerebri) [43]. In patients with subarachnomegaly, it appears that venous abnormalities leading to a post-capillary (stenotic) intracranial hypertension are more common according to published cohorts, than the variant with no phlebographic findings whose pathophysiology remains elusive (possibly corresponding to pre-capillary hyperemic variant). Based on all these findings, we argue that a venous pathology with abnormal venous outflow dynamics is the most likely origin of infant subarachnomegaly, resembling the mechanism described in adults with pseudotumor cerebri [44]. Subarachnomegaly could thus be called “pseudotumor cerebri of the infant.” However, it is not entirely clear if venous abnormalities are the primary cause or the consequence of subarachnoid widening since both are part of a pathophysiological cycle (Fig. 4).

Regardless of the initial cause for the increased venous sinus pressure, it will in turn affect CSF absorption by increasing the capillary bed pressure. Increase in ICP then forces the infant skull to expand accordingly, contributing to CSAS widening. An increased capillary bed pressure will produce, apart from increasing CSF reabsorption resistance, an increase in cerebral blood volume augmenting brain turgor. This prevents exorbitant ventricular expansion despite increased CSF pressures like in hydrocephalus, forcing ventricular CSF displacement to the CSAS. Consequently, ventricular volume will remain normal or only slightly increased, at least in an initial phase. CSF pressure in CSAS will increase accordingly and further compromise venous outflow, fostered in case of venous wall instability.

## Treatment and management

Subarachnomegaly patients are largely followed without intervention. In the majority of cases, enlargement of CSAS subsides spontaneously without apparent consequences. However, as mentioned above, this may be because long-term effects may be subtle and overlooked or not investigated systematically at all. Treatment options such as acetazolamide or shunts have not been statistically compared to the conservative approach. In few cases, where neurological or developmental delay symptoms become evident, more aggressive treatment options have been used. Some authors suggest the use of acetazolamide therapy for several weeks [28, 45]. Repeated CSF tappings have also been used as an intermediate invasive option, and for the most extreme cases, CSF shunt diversion was chosen [23]. In a cohort of 7 infants presenting with subarachnomegaly, indication for shunt diversion was decided upon intracranial pressure measurements finally treating four of them [46].

CSF diversion with shunts can help to break the vicious cycle by reducing the CSF pressure to the level of the venous pressure or even below. In the case of a sinus wall instability being the cause of the venous stenosis, the decrease of the CSF pressure below the venous sinus pressure will allow the sinus to expand again, as reported previously [23, 26]. In case of a fixed sinus aplasia or stenosis, the CSF shunt nevertheless normalizes the ICP, stops abnormal skull growth, and will support brain development at normal or close to normal ICP.

Since the CSF accumulation is mostly within the CSAS, a lumboperitoneal (LP) shunt with an overdrainage preventing mechanism seems to be, from a theoretical point of view, the best option, since it drains from the spinal subarachnoidal space, which is in direct communication with the CSAS. Furthermore, ventriculoperitoneal (VP) shunts seem to have a high rate of ventricular catheter obstruction and malfunction in these patients. The combination of high venous pressure and brain turgor drives ventricle collapse as soon as CSF is removed. In the authors’ experience, all of the few VP shunts that were implanted in subarachnomegalic infants, with proven venous sinus stenosis and developmental delay, ended up with ventricular collapse despite overdrainage prevention and, hence, converted to LP shunts. LP shunt has become our standard of care for subarachnomegalic infants with developmental delay or exorbitant head growth. A lumbar catheter obstruction or overdrainage symptoms have not been encountered so far (unpublished data).

Quantitative assessments, to decide for more aggressive treatment, are lacking as a standard guideline. No imaging features or parameters have been demonstrated as clear predictors to stratify the risk of neurodevelopmental impact. Along these lines, recent advances on MR sequences may help to clarify the relationship between parameter changes and long-term outcomes in the absence of adverted neurodevelopmental symptoms. In stratifying radiological and clinical (including invasive) findings according to the developmental status, indications for treatment will be more clear and evidence-based.

## Long-term outcome

The majority of patients are considered as neurologically and developmentally normal on follow-up [1]. However, most studies do not use thorough developmental tests; hence, subtle impairments may be underdiagnosed [8]. Nevertheless, developmental delays as mild gross motor and minimal language delay are described as transient reaching normal milestones at 2 years of age [1]. Functional long-term studies show that although the majority is within the normal range of performance, a significant proportion presents with subtle impairments as decreased attention skills and low borderline visuomotor scanning performance [47] or failure to reach gross motor function milestones [6, 19]. Pathological consequences may appear in the long run due to chronically abnormal venous and CSF dynamics after exhausting compensative mechanisms. It is unclear how these venous anomalies evolve over time in these children and its relative impact. From single observation, these consequences may attenuate or even resolve when subarachnoid collections subside either with treatment or spontaneously. Notwithstanding, increased mechanical pressure within brain tissue, as described by DTI findings, even self-limiting, may be relevant since it affects a critical developmental window and may interfere with the acquisition of certain skills.

Considering this condition as benign has prevented invasive pressure recordings, close radiological follow-up, and regular developmental assessments as a standard of care for these patients. Therefore, ICP values and dynamics for these patients still remain largely unclear and must be investigated in relation to long-term outcomes and related radiological features. Other CSF and venous dynamic-related pathologies should also be ruled out in adulthood for these subarachnomegaly patients since they may share etiological factors with known clinical pictures that express later in life. Normal pressure hydrocephalus patients, for instance, exhibit larger head circumferences compared to control subjects [48]. Furthermore, cerebrovascular flow in these NPH patients is described as abnormal, including a lower sagittal sinus venous outflow relative to the inflow [49].

## Conclusions

Subarachnomegaly is an age-related condition of the infancy with radiological enlargement of CSAS and often self-limiting course. There is increasing evidence that supports cerebrovenous system abnormalities as the main pathophysiological factor resembling an early-onset analog of pseudotumor cerebri encountered in older children and adults.

Considering the evidence on pathophysiology as outlined herein and long-term outcome reports, long-term follow-up data investigations with larger cohorts are needed to assess the consequences of venous outflow impairment and enlarged CSAS and how this relates to imaging findings and neurodevelopment test results later in life.
